# Chloroplast genomes of *Lilium lancifolium*, *L*. *amabile*, *L*. *callosum*, and *L*. *philadelphicum*: Molecular characterization and their use in phylogenetic analysis in the genus *Lilium* and other allied genera in the order Liliales

**DOI:** 10.1371/journal.pone.0186788

**Published:** 2017-10-24

**Authors:** Jong-Hwa Kim, Sung-Il Lee, Bo-Ram Kim, Ik-Young Choi, Peter Ryser, Nam-Soo Kim

**Affiliations:** 1 Department of Horticulture, Kangwon National University, Chuncheon, Korea; 2 Institute of Bioscience and Biomedical Sciences, Kangwon National University, Chuncheon, Korea; 3 Advanced Radiation Technology Institute, Korea Atomic Energy Research Institute, Sinjeong, Jeongeup, Jeonbuk, Korea; 4 Department of Agricultural Life Science, Kangwon National University, Chuncheon, Korea; 5 Department of Biology, Laurentian University, Sudbury, Ontario, Canada; 6 Department of Molecular Bioscience, Kangwon National University, Chuncheon, Korea; Università di Pisa, ITALY

## Abstract

Chloroplast (cp) genomes of *Lilium amabile*, *L*. *callosum*, *L*. *lancifolium*, and *L*. *philadelphicum* were fully sequenced. Using these four novel cp genome sequences and five other previously sequenced cp genomes, features of the cp genomes were characterized in detail among species in the genus *Lilium* and other related genera in the order Liliales. The lengths and nucleotide composition showed little variation. No structural variation was found among the cp genomes in Liliales. Gene contents were conserved among four newly sequenced cp genome in *Lilium* species, the only differences being in two pseudogenes. We identified 112 genes in 13 functional categories, 18 of which carried introns that were conserved among the species in Liliales. There were 16–21 SSR loci (>12 bp, >3 repeats) in the cp genomes in *Lilium* and the genomic locations of these loci were highly variable among the species. Average mutations were 15 SNPs per 1kb and 5 indels per 1kb, respectively, in the cp genomes of the newly sequenced four *Lilium* species. Phylogenetic classifications revealed some discrepancies between trees based on the cp genomes and previous classifications based on the morphology and geographic distributions.

## Introduction

Lilies, the plants in the genus *Lilium*, are perennial herbaceous flowering plants with over 110 species distributed widely in temperate and boreal zones in the Northern Hemisphere [1]. All lilies grow from large bulbs, plant height ranging from 50 cm to 200 cm. Because lilies bear large and showy flowers in diverse colors, which are often fragrant, many commercial cultivars have been produced by interspecific hybridization [2]. Currently lilies are the number three flowering crop after roses (*Rosa*) and mums (*Chrysanthermum*) worldwide [3].

Taxonomical classification of the genus *Lilium* has been disputed and repeatedly modified since its first botanical classification into five sections based on the morphological characters by Endlicher in 1836 [4]. In 1949, Comber divided the genus into seven sections based on 13 different morphological characteristics and germination types [5]. Although the seven-section system has been slightly modified by subsequent cytogenetic and interspecific hybridization analyses [6–7], it is basically solid with only a few species being re-assigned to different sections. Recently, Pelkonen and Pirttilä [8] reviewed the lily classifications based on the morphology, cytogenetic and molecular analyses, proposing a classification into seven sections as follows; *Martagon*, *Pseudolirium* (American group), *Archelirion* (Oriental group), *Lilium* (*Candidium* group), *Sinomartagon* (Asiatic group), *Leucolirion* (Trumpet group), and *Daurolirion* (*L*. *bulbiferum* and *Dauricum* group).

Chloroplasts are cellular organelles in photosynthetic plants and algae. The chloroplast genomes (cp genome) vary typically between 120 and 170 kb in, and are comprised of a quadripartite structure that includes two copies of invert repeat (IR) regions separated by a large-single copy (LSC) and a small-single copy (SSC) region [9–10]. The number of genes encoded in cp genome varies from 100–120 genes that are often arranged in an operon-like manner and transcribed as polycistronic precursor mRNAs which are processed into mature mRNAs by splicing and nucleolytic cleavage [10–12]. The inheritance of the cp genome is predominantly by maternal inheritance except in a few species of eudicots in the families of Geraniaceae, Campanuclaceae and Fabaceae which have biparental cp genome inheritance [10]. Because the uniparental inheritance does not allow sequence shuffling by recombination, the cp genome sequences have been the primary choice for delineating maternal lineages in plant systematic studies [13–15]. In *Lilium* and allied genera, Hayashi and Kawano [16] analyzed the phylogenetic relationships using two cp genes, *rbcL* and *matK*, sequences according to which the species in the genus *Lilium* can be grouped into three different major groups. The authors argued that the molecular-systematic results were not congruent with the classifications based on morphology. In the phylogenetic analysis of *Lilium* species endemic in Qinghai-Tibet Plateau (Q-T Plateau) using *matK* sequences, Gao et al. [17] grouped these lilies into 9 lineages in which the species in different sections of Comber [4] and Pelkonen and Pirttilä [8] were mixed. Moreover, the phylogenetic grouping using the *matK* gene sequences were different from grouping based on the nuclear ITS sequence [17].

The advent of the next-generation sequencing technology and various bioinformatics tools have allowed easier gaining of more cp genome sequences in diverse plant species [18–20]. In lilies, the whole cp genome sequences have been reported for *L*. *taliense* [20], *L*. *tsingtauense* [21], *L*. *hansonii* [22], *L*. *fargesii* [23], *L*. *cernuum* [24], *L*. *distichum* [25], *L*. *longiflorum* [26], and *L*. *superbum* (KP462883). In the present work we are adding four more *Lilium* species with a sequenced whole cp genome; *L*. *amabile*, *L*. *callosum*, *L*. *lancifolium*, and *L*. *philadeliphicum*. The four species were chosen to add the chloroplast genomes in the Korean endemic *Lilium* species in the section *Sinomartagon* and compare them with the cp genome of *L*. *philadelphicum* that is a native North American species in the section *Pseudolilium* [8]. The current report contains the comprehensive genomic and phylogenomic analyses of the cp genomes in the genus *Lilium*.

## Materials and methods

### DNA preparations, sequencing, and assembly

Chloroplast genomes of four *Lilium* species were sequenced: *L*. *lancifolium*, *L*. *amabile*, *L*. *callosum* and *L*. *philadelphicum*. *L*. *lancifolium* (Accession No GWL0702), *L*. *amabile* (Accession No GWL15789), and *L*. *callosum* (Accession No GWL3662) were accessions that have been maintained at the *Lilium* germplasm nursery in Kangwon National University, Korea. *L*. *philadelphicum* was an accession collected from its natural habitat (46° 2' 5.63"N; 81° 46' 23.172" W) close to Sudbury, Ontario, Canada, in June 2016. *L*. *philadelphicum* is not on the list of the endangered or protected species, and no permissions were required for collections of leaves for this specimen from its natural habitats.

Fresh leaves (~100 mg) were sampled from young plants. Cellular DNA was extracted using the DNAeasy Plant Maxi Kit (QIAGEN, Valencia, CA, USA). DNA (5 ug) samples were then sheared to an average size about 300 bp by nebulization with compressed N_2_ gas. Quality of the sheared DNA was assessed using a Bioanalyzer 2200 (Agilent Technologies, Santa Clara, CA, USA), and a paired-end library was constructed using the Illumina Paired-End Library Kit (Illumina, San Diego, CA, USA). Genomic DNA sequencing was then carried out on a single lane of a HighSeq 2000 flow cell by Phyzen Inc. (Seoul, Korea). The sequence was filtered and assembled using *de novo* assembly package software, CLC Assembly Cell v.4.2.1 (https://www.qiagenbioinformatics.com/products/clc-assembly-cell/, Quigen Co., Ltd. Hilden, Germany) for a complete chloroplast genome assembly using the dnaLCW method (*de novo* assembly of low coverage whole-genome shotgun sequencing method) as suggested protocol of Kim et al. [27]. The ambiguous sequences including structural borders and mono-polymer were manually edited. The complete chloroplast genome map was produced using reported chloroplast genomes from other *Lilium* species as references (KM103364 in *L*. *hasonii*, KC968977 in *L*. *longiflorum*, KX592156 in *L*. *fargesii*, KP462883 in *L*. *superbum*) [20–26]. The circular chloroplast genome map was then drawn using the OrganellarGenomeDRAW tool (ORDRAW) [28].

### Gene and simple sequence repeat (SSR) annotation

Gene annotation of the newly sequenced cp genomes was performed using the Dual Organellar GenoMe Annotator (DOGMA) [29], and all initiation and stop codons were manually confirmed in the DOGMA-annotated data. Predicted introns were further checked by comparison with other cp genome sequences, and all annotated transfer RNA (tRNA) genes were verified using ARAGORN [30]. SSR sequences were detected with the UGENE program (http://ugene.net/) by a command “Find tandems” with a default set a minimum size 12 bp and repeat count 3.

### SNPs/Indel analysis

The nine cp genome sequences were aligned using MAFFT version 7 program (http://mafft.cbrc.jp/alignment/software/). The VCF (variant call format) was built using Msa2vcf (http://lindenb.github.io/jvarkit/MsaToVcf.html). Then, the SNPs and indels were identified manually.

### Sequence identity and phylogenetic analysis among the cp genomes in Liliales

Cp genomes of 13 species in the order Liliales (nine *Lilium* species, two *Fritillaria* species, one of each *Smilax* and *Alstroemeri*a species) were used for sequence identity and phylogenetic analyses. The cp genome of *Allium cepa* (order Asparagales) was used as an out-group in the analyses. Except for the four newly sequenced cp genomes, the cp genomes were downloaded from GenBank. A multiple sequence alignment was then generated in ClustalW, and gaps were edited using the MEGA5 program [31]. For sequence identity comparison and sequence divergence along the cp genomes, sequences were compared and plotted using the mVISTA program (http://genome.lbl.gov/vista/mvista/submit.shtml). For phylogenetic analyses, two data sets were used; one with the whole cp genome sequences and another with protein coding sequences. After maximum parsimony analysis was performed with PAUP v4b10 [32], maximum likelihood (ML) analyses were performed with 1000 bootstrap replicates using RAxML-HPC BlackBox v.8.1.24 at Cipres Science Gateway site (http://www.phylo.org/tools/obsolete/raxmlhpc2.html#) [33].

## Results

### Cp genome length and AT contents among the *Lilium* species

**Fig 1:**
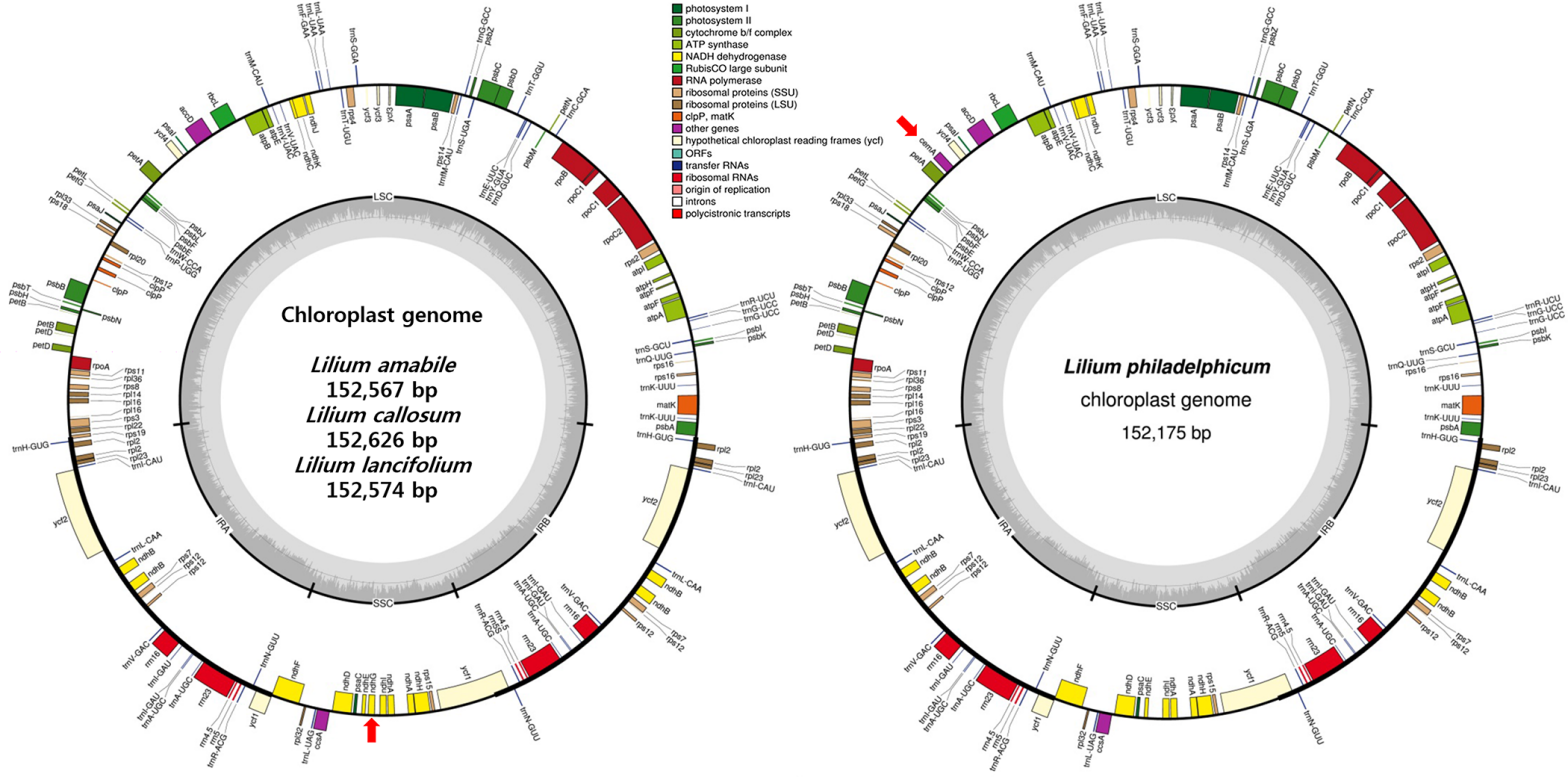
Chloroplast genome maps of four *Lilium* species. The gene orders of the cp genomes of *L*. *amabile*, *L*. *callosum*, and *L*. *lancifolium* were identical but different from the cp genome of *L*. *philadelphicum* by two pseudogenes (red arrows). The former three cp genomes have a pseudogene *ndhG* in SSC region, but this pseudo gene was absent in *L*. *philadelphicum*. The pseudogene *cemA* in LSC was present in the cp genome in *L*. *philadelphicum*, but absent in the former three cp genomes.

The complete cp genomes of four *Lilium* species were successfully assembled using high-quality Illumina sequence data filtered by CLC Assembly Cell software. The cp genomes were assembled with average coverage depth 177x in *L*. *amabile*, 92x in *L*. *callosum*, 58x in *L*. *lancifolium*, 116x *L*. *philadelphicum*, respectively, using at least 13 Gbp genome sequence data generated by Illumina sequencer platform (S1 Table). Table 1 summarizes the length of cp genomes and GC contents in *Lilium* species. Total lengths of the cp genomes range from 152,175 in *L*. *philadelphicum* to 153,235 in *L*. *fargesii*. The lengths of LSC range from 81,580 in *L*. *philadelphicum* to 82,230 in *L*. *longiflorum*, and those of SSC from 17,038 in *L*. *fargesii* to 17,620 in *L*. *hansonii*, respectively. The lengths of IRs varies from 26,491 in *L*. *callosum* to 26,990 in *L*. *fargesii*. The nucleotide compositions of cp genomes had a high AT content in the range of 62.93% in *L*. *philadelphicum* to 63.01% in *L*. *fargesii*. The IR regions showed lower AT ratio than the LSC and SSC regions in all *Lilium* species. Thus, the length and nucleotide variations were low among the cp genomes in the *Lilium* species. The four newly sequenced cp genomes in the current study did not show any structural and gene order variations (Fig 1). The cp genomes were deposited to GenBank with accession numbers KY940844 for *L*. *lancifolium*, KY940845 for *L*. *amabile*, KY940846 for *L*. *callosum*, and KY940847 for *L*. *philadelphicum*, respectively.

**Table 1:** Chloroplast genome length and A+T contents among eight *Lilium* species.

species	*L*. *ama*	*L*. *call*	*L*. *lan*	*L*. *phil*	*L*. *han*	*L*. *long*	*L*. *far*	*L*.*dis*
Total length (bp)	152,567	152,626	152,574	152,175	152,655	152,793	153,235	152,598
LSC (bp)	82,001	82,040	82,005	81,580	82,051	82,230	82,217	82,031
SSC (bp)	17,582	17,604	17,585	17,521	17,620	17,523	17,038	17,487
IRs (bp)	26,492	26,491	26,492	26,537	26,492	26,520	26,990	26,540
% of AT								
LSC	65.16	63.92	65.15	65.07	65.18	62.98	65.28	65.08
SSC	69.26	69.30	69.26	69.41	69.31	69.21	69.26	69.32
IRs	57.52	57.51	57.52	57.50	57.52	57.56	57.80	57.52
Total	62.97	62.99	62.97	62.93	63.00	62.98	63.01	62.94

### Genes encoded in the cp genomes in lilies

In each cp genome of the four newly sequenced *Lilium* species, we annotated a total of 156 genes, of which 102 are protein-coding genes, 46 are tRNA genes, and 8 are ribosomal RNA (rRNA) genes (S2 Table). Because some genes are duplicated or triplicated, the 156 genes are classified into 112 different genes. Table 2 shows the 112 genes that are classified into 13 functional categories, with no differences among the four newly sequenced cp genomes. The LSC and SSC regions contain 96 and 12 genes, respectively, and each IR region has 24 genes that are inversely oriented to one another. There are two pseudogenes, *ndhG* in *L*. *philadeliphicum* and *cemA* in *L*. *amabile*, *L*. *callosum* and *L*. *lancifolium*, which carried premature stop codons (Table 2).

**Table 2:** Gene products of the cp genomes in *L*. *amabile*, *L*. *callosum*, *L*. *lancifolium* and *L*. *philadelphicum*.

Photosystem I	psaA, B, C, I, J, ycf3^2)^, ycf4
Photosystem II	psbA, B, C, D, E, F, H, I, J, K, L, M, N, T, Z
Cytochrome b6/f	petA, B^1)^, D^1)^, G, L, N
ATP synthase	atpA, B, E, F^1)^, H, I
Rubisco	rbcL
NADH oxidoreductase	ndhA^1)^, B^1)^, C, D, E, F, G^5)^, H, I, J, K
Large subunit ribosomal proteins	rpl2^1)^ ^3)^, 14, 16^1)^, 20, 22, 23^3)^, 32, 33, 36
Small subunit ribosomal proteins	rps2, 3, 4, 7^3)^, 8, 11, 12 ^1)^ ^3)^ ^4)^, 14, 15, 16^1)^, 18, 19^3)^
RNA polymerase	rpoA, B, C1^1)^, C2
Unknown function protein coding gene	ycf1, 2^3)^
Other genes	accD, ccsA, cemA^6)^, clpP^2)^, matK
Ribosomal RNAs	rrn16^3)^, 23^3)^, 4.5^3)^, 5^3)^
Transfer RNAs	trnA-UGC^1)^^3)^, trnC-GCA, trnD-GUC, trnE-UUC, trnF-GAA, trnfM-CAU, trnG-GCC, trnG-UCC^1)^, trnH-GUG^3)^, trnI-CAU^3)^, trnI-GAU^1)^^3)^ trnK-UUU^1)^, trnL-CAA^3)^, trnL-UAA^1)^, trnL-UAG, trnM-CAU, trnN-GUU^3)^, trnP-UGG, trnQ-UUG, trnR-ACG^3)^, trnR-UCU, trnS-GCU, trnS-GGA, trnS-UGA, trnT-GGU, trnT-UGU, trnV-GAC^3)^, trnV-UAC^1)^^,^ trnW-CCA, trnY-GUA

^1)^ Gene containing a single intron

^2)^ Gene containing two introns

^3)^ Two gene copies in IRs

^4)^ Trans-splicing gene

^5)^ Pseudogene in *L*. *philadelphicum*

^6)^ Pseudogene in *L*. *amabile*, *L*. *callosum* and *L*. *lancifolium*

Eighteen genes contain introns; ten protein-coding genes (*rps16*, *atpF*, *rpoC1*, *petB*, *petD*, *rpl16*, *rpl2*, *ndhB*, *rps12*, *ndhA*) and six tRNA genes (*trnK-UUU*, *trnG-UCC*, *trnL-UAA*, *trnV-UAC*, *trnI-GAU*, *trnA-UGC*) have single introns, whereas two protein-coding genes (*clpP* and *ycf*3) have two introns each. One intron-containing gene (*rps12*) is trans-splicing, having the first exon in the LSC and the second and third exons in IR regions (Table 2).

Of the 18 intron-containing genes, introns in 17 genes were conserved among the species in the genera *Lilium*, *Fritillaria*, and *Smilax* in the order Liliales (Table 3). The intron in *trnG-UCC* was not present in the *L*. *fargesii* and two *Fritillaria* species. Six genes including the *trnG-UCC* showed intron absence in *Allium cepa* in the order Asparagales.

**Table 3:** Presence or absence of introns in 18 genes in 13 species in the order Liliales and *Allium cepa*.

Genes	*L*. *am*	*L*. *ca*	*L*. *la*	*L*. *ph*	*L*. *ha*	*L*. *lo*	*L*. *su*	*L*. *di*	*L*. *fa*	*F*. *ci*	*F*. *ta*	*S*. *ch*	*A*. *au*	*A*. *ce*
petB	o	o	o	o	o	o	o	o	o	o	o	o	o	-
petD	o	o	o	o	o	o	o	o	o	o	o	o	o	-
rpl2	o	o	o	o	o	o	o	o	o	o	o	o	o	o
rpl16	o	o	o	o	o	o	o	o	o	o	o	o	o	-
rps16	o	o	o	o	o	o	o	o	o	o	o	o	o	-
atpF	o	o	o	o	o	o	o	o	o	o	o	o	o	o
rpoC1	o	o	o	o	o	o	o	o	o	o	o	o	o	o
ndhA	o	o	o	o	o	o	o	o	o	o	o	o	o	o
ndhB	o	o	o	o	o	o	o	o	o	o	o	o	o	o
ycf3	o	o	o	o	o	o	o	o	o	o	o	o	o	o
rps12	o	o	o	o	o	o	o	o	o	o	o	o	o	-
clpP	o	o	o	o	o	o	o	o	o	o	o	o	o	o
trnA-UGC	o	o	o	o	o	o	o	o	o	o	o	o	o	o
trnG-UCC	o	o	o	o	o	o	o	o	-	-	-	o	o	-
trnI-GAU	o	o	o	o	o	o	o	o	o	o	o	o	o	o
trnK-UUU	o	o	o	o	o	o	o	o	o	o	o	o	o	o
trnL-UAA	o	o	o	o	o	o	o	o	o	o	o	o	o	o
trnV-UAC	o	o	o	o	o	o	o	o	o	o	o	o	o	o

Note: *L am*; *L*. *amabile*: *L*. *ca*; *L*. *callosum*: *L la*; *L*. *lancifolium*: *L*. *ha*; *L*. *hannai*: *L*. *su*; *L*. *superbum*: *L*. *di*; *L*. *distichum*: *L*. *fa*; *L*. *fargesii*: *L*. *ci*; *L*. *cirrnum*: *F*. *ci*; *Fritillaria cirrhosa*: *F*. *ta*; *F*. *taipaiensis*: *S*. *ch*; *Smilax china*: *A*. *au*; *Altroemeria aurea*: *A*. *ce*; *Allium cepa*.

### SSR sequences in the cp genomes in *Lilium* species

We identified 96 SSR loci with a threshold of over 10 bp and 3 repeats and the 96 SSR loci consisted of 14 di-nucleotide repeats, 74 tri-nucleotide repeats, and 8 tetra-nucleotide repeats in *L*. *lancifolium* cp genome (data not shown). When the stringency was increased to a threshold over 12 bp and 3 repeat count, the number of SSR loci was narrowed to 42 SSR loci which consisted of eight di-nucleotide repeats, 12 tri-nucleotide repeats, 17 tetra-nucleotide repeats, and five penta-nucleotide repeats (Table 4). The SSR loci were mostly present in the LSC regions except of the three loci in SSC. No SSR locus was present in the invert repeat regions (IRs). Twelve, three, and 27 SSR loci were present in intronic regions, exons and intergenic spacers, respectively. The number of SSR loci varied from 16 in *L*. *lancifolium* to 21 in *L*. *fargesii* and the presence/absence polymorphisms were highly variable among the species. Of the 42 SSR loci, only four loci were present in all the *Lilium* cp genomes. *L*. *amabile* and *L*. *callosum* shared exact SSR loci and repeat numbers. The SSR loci in *L*. *lancifolium* were all present in *L*. *amabile* and *L*. *callosum*, but one locus (*trnL-UAA*) at LSC was different in the number of repeats as (AT)_8_ in *L*. *lancifolium* and (AT)_10_ in *L*. *amabile* and *L*. *callosum*

**Table 4:** Distribution of SSR sequences in the cp genomes of *Lilium* species.

SSR type	*L*. *am*	*L*.*ca*	*L*.*la*	*L*.*ph*	*L*.*ha*	*L*.*lo*	*L*.*su*	*L*.*fa*	*L*.*di*	
AT	-	-	-	-	-	-	-	6	6	LSC(trnK-UUU)*
AT	6	6	6	7	6	6	6	6	-	LSC(trnS-GCU- trnG-UCC)***
AT	-	-	-	-	-	6	-	-	-	LSC(trnS-GCU- trnG-UCC)***
AT	-	-	-	-	-	-	6	-	6	LSC(rpoB-trnC-GCA)***
AT	-	-	-	-	-	6	6	7	6	LSC(trnL-UAA)*
AT	-	-	-	-	-	6	-	-	-	LSC(petB-petD)***
AT	10	10	8	-	8	-	-	-	-	LSC(trnL-UAA)*
AC	-	-	-	-	-	-	6	-	-	LSC(psbK-psbI)***
AAT	-	-	-	-	4	-	-	-	-	LSC(accD-psaI)***
ATA	4	4	4	-	4	4	-	4	4	LSC(petD-rpoA)***
ATT	-	-	-	-	-	4	-	-	-	LSC(ycf1)**
TTG	-	-	-	-	-	6	-	-	-	LSC(matK-rps16)***
TTG	4	4	-	-	-	-	-	-	-	LSC(rps16)*
TAT	4	4	4	4	4	-	4	-	4	LSC(trnT-UGU-trnL-UAA)***
TAT	-	-	-	-	-	-	4	4	4	LSC(petD-rpoA)***
TTA	4	4	4	4	4	4	4	4	4	LSC(trnV-UAC)*
TTA	-	-	-	4	-	-	-	-	-	LSC(psaJ-rpl33)***
TTA	4	4	4	4	5	4	-	-	-	SSC(rps15-ycf1)***
GAA	4	4	4	4	4	4	4	4	4	LSC(accD-psaI)***
AAAT	-	-	-	-	-	3	-	-	-	LSC(rps16-rtnQ-UUG)***
AAAT	-	-	-	4	-	-	-	-	-	LSC(psbM-trnD-GUC)***
AAAT	-	-	-	-	-	-	-	3	3	LSC(psaJ-rpl33)***
AAAT	4	4	4	3	4	3	3	3	4	SSC(ndhG-ndhI)***
AATA	3	3	3	3	3	3	3	3	3	LSC(rpl22)**
AATA	3	3	3	3	3	3	3	3	3	SSC(ndhD)**
AATT	3	3	3	3	3	3	3	3	3	LSC(atpI-rps2)***
ATTT	-	-	-	-	-	-	-	-	3	LSC(rpoC1)*
AGAA	-	-	-	-	-	-	-	3	3	LSC(trnS-GCU-trnR-UCU)***
AGAA	3	3	3	3	3	3	3	-	-	LSC(trnG-UCC-trnR-UCU)***
TTTA	3	3	3	3	3	3	3	3	-	LSC(rpoC1)*
TTTA	3	3	3	3	3	3	-	-	-	LSC(rpoC1)*
TTTA	-	-	-	3	-	-	4	3	3	LSC(psaA-ycf3)***
TTAT	-	-	-	3	-	-	3	3	3	LSC(ycf3)*
TTAT	-	-	-	3	-	-	-	3	3	LSC(trnT-UGU-trnL-UAA)***
TTCT	-	-	-	3	-	-	3	3	3	LSC(rpl16)*
TAAT	3	3	3	-	3	3	-	-	-	LSC(psbM-trnD-GUC)***
AAATA	-	-	-	-	-	-	-	3	-	LSC(atpH-atpI)***
AATTA	-	-	-	-	-	3	-	-	-	LSC(rbcL-accD)***
AAATA	-	-	-	-	-	-	3	3	-	LSC(atpH-atpI)***
TTTAC	3	3	3	-	3	-	-	3	-	LSC(rpoC1)*
TTAAG	-	-	-	-	3	-	-	-	3	LSC(accD-psaI)***

* Intron region

** Coding region

*** Intergenic region

### SNPs and Indels among cp genomes in *Lilium* species

**Fig 2:**
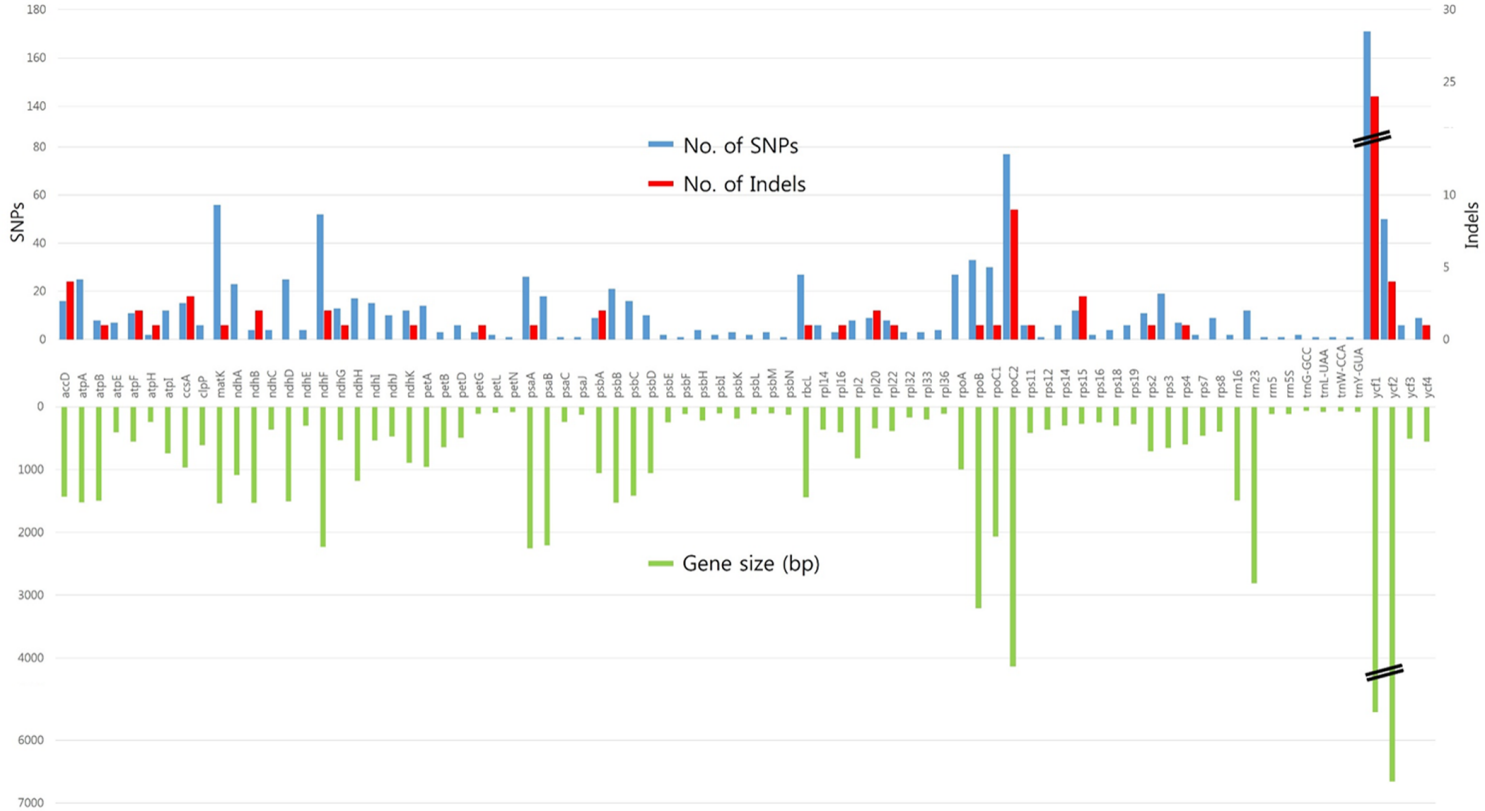
Numbers of SNPs and indels in 82 genes among nine cp genomes in *Lilium* species.

We identified 3,018 mutations which consisted of 2,271 SNPs and 747 indels among the 4 newly sequenced cp genomes (Table 5, S3 Table). The average variations were 15 SNPs per 1 kb and 5 indels per 1kb, respectively. The most variable region was in the introns with 67.7 mutations per 1 kb, followed by the intergenic region with 36 mutations per 1 kb. Of the 112 genes, 80 genes showed variations (Fig 2, S4 Table). Of the 80 genes with SNPs, only 27 had indels. The number of SNPs in a gene was not related with the number of indels, 19 genes having more SNPs than indels, while 7 genes had more indels than SNPs (S5 Table). Gene length was highly correlated with the number of SNPs, but the the number of indels was not related with the gene length. Four of the 46 tRNA genes showed variations.

**Table 5:** Numbers of SNPs and indels in different regions of cp genomes in *L*. *amabile*, *L*. *callosum*, *L*. *lancifolium* and *L*. *philadelphicum*.

	Intergenic	Genic		Total
	51,365 bp	Exon	Intron	152,567 bp
		84,026 bp	17,176 bp	
No. of SNPs	1,305	966	1,047	2,271
Frequency (%)	2.54	1.15	6.1	1.49
No. of InDels	558	73	116	747
Frequency (%)	1.09	0.09	0.68	0.49
Total	1,863	1,039	1,163	3,018
Frequency (%)	3.63	1.24	6.77	1.98

### Sequence divergence along the cp genomes among species in Liliales

**Fig 3:**
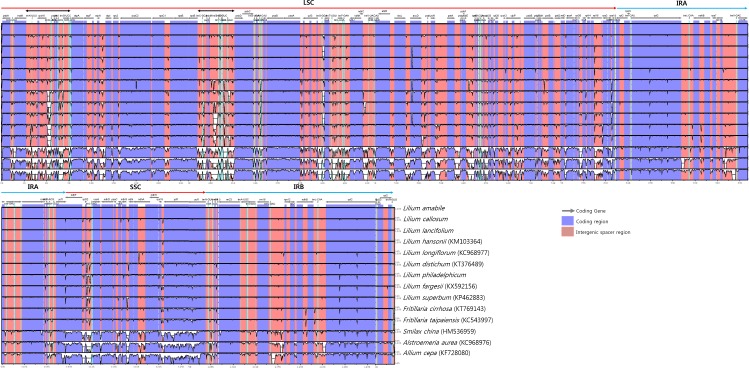
Sequence identity plots among 13 species in the order Liliales and *Allium cepa*.

We identified no major structural variations such as inversions or large deletions in cp genomes of the 9 *Lilium* species. Sequence divergence hotspot regions along the cp genomes were analyzed among nine *Lilium* species. Five other species (two *Fritillaria* species, *Smilax china*, *Alsroemeria aurea*, and *Allium cepa*) were included in the cp genome variation survey (Fig 3). Among the *Lilium* species, most sequence variations were found in the noncoding intergenic regions in the LSC and SSC regions. Two hypervariable regions were identified in the gene-sparse intergenic regions in LSC, and are designated by bars at the top of Fig 3. The sequence variations in the IR regions were comparably lower than the LSC and SSC regions. In comparisons beyond the Liliales, sequence variations were also present in intergenic regions throughout the cp genomes. As expected, sequence divergence among the species in Liliaceae (the genera *Lilium* and *Fritillaria*) was lower along the whole cp genomes, compared to the divergence among all the species.

### Phylogenetic analysis among species in Liliales

**Fig 4:**
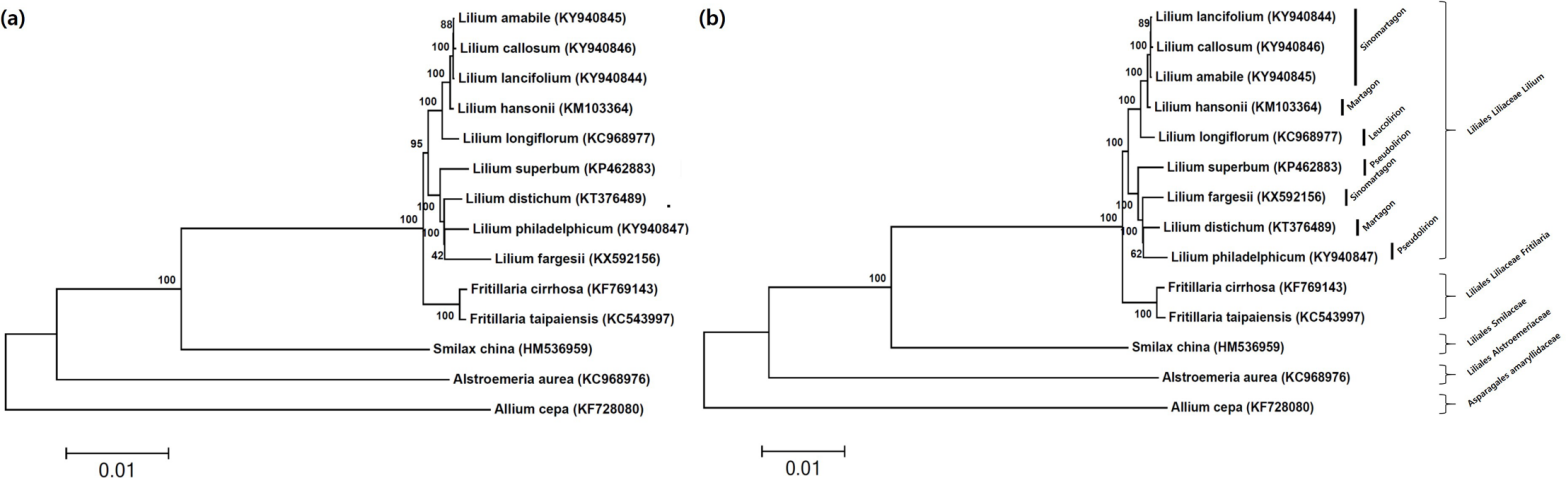
Phylogenetic trees based on the whole cp genome sequences (A) and functional genes (B) among 13 species in the order Liliales and *Allium cepa*. The trees were made using maximum likelihood algorithm and the numbers on the nods designate the bootstrap values.

Phylogenetic trees based on the whole-cp genomes and those based on the 71 protein coding genes were not different from each other, and showed clustering which agreed with the taxonomical hierarchical order (Fig 4). *Allium cepa* in the order Asparagales was out-clustered from the species in Liliales. Among the species in Liliales, *Alstroemeria aurea* in the family Alstroemeriacea and *Smilax china* in the family Smilaceae were out-grouped from the Liliaceae species. The two *Fritillaria* species showed distinct clustering from the species in the genus *Lilium*. The nine *Lilium* species were clustered in two groups; one group with three Sinomartagon lilies (*L*. *lancifolium*, *L*. *callosum*, and *L*. *amabile*), one Martagon lily (*L*. *hansonii*), and one Leucolirion lily (*L*. *longiflorum*), and another group with two Pseudolirium lilies (*L*. *superbum* and *L*. *philadelphicum*), one Sinomartagon lily (*L*. *fargesii*), and one Martagon lily (*L*. *distichum*).

## Discussion

This report contains novel cp genome sequences of four *Lilium* species and other previously sequenced cp genomes in Liliales for the purpose of genomics and phylogenomics analyses, based on the whole cp genome sequences. The cp genomes in nine *Lilium* species, including five previously sequenced cp genomes showed remarkably low variations in length, base compositions, gene contents, intron retentions, and genome structure. Cp genomes of certain lineages of land-plants have undergone gene losses and significant structural rearrangements [34]. A good example is the genus *Astragalus* in the family Fabaceae, in which inversions and gene losses resulted in the variations in cp genome structure and gene contents between species [19]. In the analysis of 81 genes from 64 plastid genomes, Jansen et al. [34] reported 62 independent gene and intron losses that are limited to more derived monocot and eudicot clades. Kim and Kim [26] surveyed gene losses among cp genomes in monocots and noted that gene losses were frequent events in some monocot families. Among three families, Liliaceae, Smilaceae, and Alstromeriaceae in the order Liliales, they found that gene content and order were conserved except of the *infA* loss in *Smilax* and *Altroemeria*. Introns in cp genes were known to be generally conserved in land-plant cp genomes. We observed an intron loss polymorphism in *trnG-UCC* gene among the *Lilium* speices and two *Frillaria* species. This intron, however, was present in *Smilax china* and *Alstromemeria aurea* in Liliales, but absent in *Allium cepa* in Asparagales. The presence/absence polymorphism of this gene was also reported both among monocot and eudicot species [34]. Thus, the intron loss of this gene must have happened independently, rather than in a lineage specific manner.

Simple sequence repeats (SSR) occur in both nuclear and cp genomes in all plants. Cp SSRs have been demonstrated as robust marker systems in population genetics and ecology [35–37], but has some drawbacks due to low variation compared to the high polymorphism in nuclear SSRs [38]. Prior to this report, several cp genomes in *Lilium* species have been reported [20–26], but no data on the cp SSRs are available. SSRPs (simple sequence repeat polymorphisms) are derived from two mechanisms such as unequal crossing-over and DNA replication slippage [39]. However, there is no unequal crossing-over in the cp genome SSRs, resulting in the low intra-specific polymorphisms as noted by Wheeler et al. [38]. Because once the SSR sequences occur *de novo* in the cp genome, they may stay in the position in the lineages. Thus, the presence/absence polymorphisms of the SSR locus between species may be useful indicators in the analysis of genetic relatedness. In practice, *L*. *amabile* and *L*. *callosum*, shared the exact loci, these two species also showed a very close phylogenetic relatedness.

Cp genome structural changes have been noted in several unrelated lineages in flowering plants such as Geraniaceae [40], Onagraceae [41], Campanulaceae [42], and Fabaceae [43]. Inversions and heteroplasmic variations have been reported within the genus *Astragalus* in the family Fabaceae [19]. However, no structural variations were observed among the cp genomes in the genus *Lilium* in the current study. Conservation of the cp genome structure in Liliales has also been reported by Kim and Kim [26], supporting our finding of constrained structural variation in the cp genomes in the genus *Lilium*. In a comparison between two cp genomes of tropical trees in the genus *Machilus* in the family Lauraceae, Song et al. [44] counted 297 mutation events including a micro-inversion, 65 indels, and 231 substitutions. In the coding regions, they counted 95 SNPs between the two species. The number mutations in the cp genomes in *Lilium* species observed in the current study was comparatively higher. The discrepancy between the two studies may derive from the difference in the number cp genomes: four cp genomes in our study compared to two cp genomes in the study by Song et al [44].

We identified two hypervariable regions in the LSC regions. Zhang et al. [20] surveyed the mutations in cp genome wide variations in five *Epimedium* species in the family Berberidaceae, in which overall variation patterns along the cp genomes are congruent with our results, but they did not observe such prominent hypervariable. In our analysis, the two hypervariable regions were also found in the *Fritillaria* species in Liliaceae. Shaw et al. [14, 45–46] surveyed noncoding cp DNA sequences among angiosperm species to choose the regions for phylogenetic and phylogeographic studies, in which they showed that most variations are in the noncoding intergenic regions in LSC and SSC regions. Moreover, they reported two variable regions within the LSC and one within the SSC. The two hypervariable regions in our study were the same regions as in their report in LSC. However, *Smilax* and *Alstoemeria* species in the order Liliales do not have the conspicuous hypervariable regions which show variations along the LSC and SSC regions. Thus, the two hypervariable regions might be limited to the Liliaceae or to the tribe Lilieae.

Cp genome sequences have been employed for phylogenetic analysis in the genus *Lilium* by several investigators [20–24, 26]. We are adding four novel cp genomes to have more comprehensive analyses on interspecific relationships. Our analyses basically confirms the phylogenetic trees based on the whole cp genome sequences and protein coding genes. The nine *Lilium* species were clustered into two groups in the phylogenetic trees (Fig 3), which was consistent with the sequence divergence patterns generated by the mVISTA program (Fig 2). Our results are congruent with the results of Bi et al. [23]. In their study, seven *Lilium* cp genomes were grouped into two groups in which the *L*. *superbum* (section *Pseudolirion)* and *L*. *fargesii* (section *Sinomartagon*) were grouped into one cluster and *L*. *longiflorum* (section *Leucolirion*) and *L*. *hansonii* (section *Martagon*) into another. However, the cp genome-based phylogenetic trees are incongruent with recent classification of the morphological features and geographic origin [8]. This was also reported by Hayashi and Kawano [16] in their study of phylogenetic relationships based on two cp genes, *rbcL* and *matK*, among *Lilium* species and related genera. Gao et al. [17] also noted that the phylogenetic groupings were dissimilar among the *Lilium* species collected from Q-T plateau in China based on the nuclear ITS and cp *matK* sequence variations. The phylogenetic relationship inferred from retrotransposon based markers showed the *L*. *lancifolium* in *Sinomartagon* was not grouped with *L*. *callosum* and *L*. *amabile* in *Sinomartagon* section [47]. The two *Martagon* lilies, *L*. *hansonii* and *L*. *distichum* were clustered in the same group in their report, but these two species were separated into different groups in our study. The high bootstrap values indicate the robustness in the current analysis. Thus, the discrepancies might be derived from the phylogenetic inferences from maternal inheritance of cp genomes and biparental inheritance of nuclear genomes.

## Conclusion

The comparative genomic and phylogenomic analyses of the cp genomes in the genus *Lilium* and other related genera in the order Liliales revealed high conservation in length, AT ratios, gene contents and genome structures. There were 18 intron-containing genes. One intron loss was observed in species- relationship independent manner. We observed 16–21 SSR loci and high variations of presence/absence polymorphisms among the cp genomes among the species in the genus *Lilium*. Compared to the limited length and structure variations, there were significant numbers of sequence variations of SNPs, indels and SSR loci in the cp genomes of the genus *Lilium*. The two hyper-variable regions in the LSC may need to be compared with cp genomes of other distantly related genera for a better understanding of selection constraints along the cp genomes. Discrepancies in the positions of some species in the phylogenetic trees should be further analyzed. The presence/absence polymorphisms in SSR loci in the cp genomes may be expanded to more species to trace for the maternal lineages, as the SSRs stay in the current loci after *de novo* occurrence.

## Supporting information

S1 Table The data summary of genome sequence reads generated by Illumina sequencer platform and mapped sequence reads to cp genome de novo assembly. (XLSX)

S2 Table The list of genes encoded in the cp genomes in *L. amabile*, *L. callosum*, *L. lancifolium* and *L. philadelphicum*. (XLSX)

S3 Table Number of SNPs and indels in 80 genes in *L. amabile*, *L. callosum*, *L. lancifolium* and *L. philadelphicum*. (XLSX)

S4 Table The SNPs and their locations in the cp genomes in nine *Lilium* species. (XLSX)

S5 Table The indels and their locations in the cp genomes in nine *Lilium* species. (XLSX)
